# APOBEC3 Interference during Replication of Viral Genomes

**DOI:** 10.3390/v7062757

**Published:** 2015-06-11

**Authors:** Luc Willems, Nicolas Albert Gillet

**Affiliations:** 1 Molecular and Cellular Epigenetics, Interdisciplinary Cluster for Applied Genoproteomics (GIGA) of University of Liège (ULg), B34, 1 avenue de L’Hôpital, Sart-Tilman Liège 4000, Belgium; E-Mail: luc.willems@ulg.ac.be; 2 Molecular and Cellular Biology, Gembloux Agro-Bio Tech, University of Liège (ULg), 13 avenue Maréchal Juin, Gembloux 5030, Belgium

**Keywords:** viral replication, quasi-species, hypo-mutation, hyper-mutation, APOBEC3, cytidine deaminase

## Abstract

Co-evolution of viruses and their hosts has reached a fragile and dynamic equilibrium that allows viral persistence, replication and transmission. In response, infected hosts have developed strategies of defense that counteract the deleterious effects of viral infections. In particular, single-strand DNA editing by Apolipoprotein B Editing Catalytic subunits proteins 3 (APOBEC3s) is a well-conserved mechanism of mammalian innate immunity that mutates and inactivates viral genomes. In this review, we describe the mechanisms of APOBEC3 editing during viral replication, the viral strategies that prevent APOBEC3 activity and the consequences of APOBEC3 modulation on viral fitness and host genome integrity. Understanding the mechanisms involved reveals new prospects for therapeutic intervention.

## 1. APOBEC3s Edit Single-Stranded DNA

The APOBEC3 enzymes are deaminases that edit single-stranded DNA (ssDNA) sequences by transforming deoxycytidine into deoxyuridine [1,2,3]. APOBEC3s are involved in the mechanisms of innate defense against exogenous viruses and endogenous retroelements [3]. The human genome codes for seven APOBEC3 genes clustered in tandem on chromosome 22 (namely A3A, A3B, A3C, A3DE, A3F, A3G, and A3H) and surrounded by the CBX6 and CBX7 genes. All APOBEC3 genes encode a single- or a double-zinc-coordinating-domain protein. Each zinc-domain belongs to one of the three distinct phylogenic clusters termed Z1, Z2 and Z3. The seven APOBEC3 genes arose via gene duplications and fusions of a key mammalian ancestor with a CBX6-Z1-Z2-Z3-CBX7 locus organization. Aside from mice and pigs, duplications of APOBEC3 genes have occurred independently in different lineages: humans and chimpanzees (*n* = 7), horses (*n* = 6), cats (*n* = 4), and sheep and cattle (*n* = 3) [4,5]. Read-through transcription, alternative splicing and internal transcription initiation may further extend the diversity of APOBEC3 proteins.

APOBEC3s are interferon-inducible genes [6] that are highly expressed in immune cells despite being present in almost all cell types [7,8]. The sub-cellular localization differs between the APOBEC3s isoforms: A3DE/A3F/A3G are excluded from chromatin throughout mitosis and become cytoplasmic during interphase, A3B is nuclear and A3A/A3C/A3H are cell-wide during interphase [9].

APOBEC3s exert an antiviral effect either dependently or independently of their deaminase activity. The deaminase activity involves the removal of the exocyclic amine group from deoxycytidine to form deoxyuridine. This process can generate different types of substitutions. First, DNA replication through deoxyuridine leads to the insertion of a deoxyadenosine, therefore causing a C to T transition. Alternatively, Rev1 translesion synthesis DNA polymerase can insert a C in front of an abasic site that is produced through uracil excision by uracil-DNA glycosylase (UNG2) leading to a C-to-G transversion [10]. In addition to inducing deleterious mutations in the viral genome, deamination of deoxycytidine can also initiate degradation of uracilated viral DNA via a UNG2-dependent pathway [11,12]. On the other hand, deaminase-independent inhibition requires binding of APOBEC3s to single-stranded DNA or RNA viral sequences at various steps of the replication cycle [13,14,15,16,17,18,19,20,21,22,23].

## 2. APOBEC3 Edition during Viral Replication Cycles

The mechanism of APOBEC3s inactivation is dependent on the type of virus and its mode of replication.

### 2.1. Retroviruses

Retroviruses are plus-strand single-stranded RNA viruses replicating via a DNA intermediate generated in the cytoplasm by reverse transcription. Human retroviruses notably include HIV (human immunodeficiency virus) and HTLV (human T-lymphotropic virus).

#### 2.1.1. HIV-1

**Figure 1:**
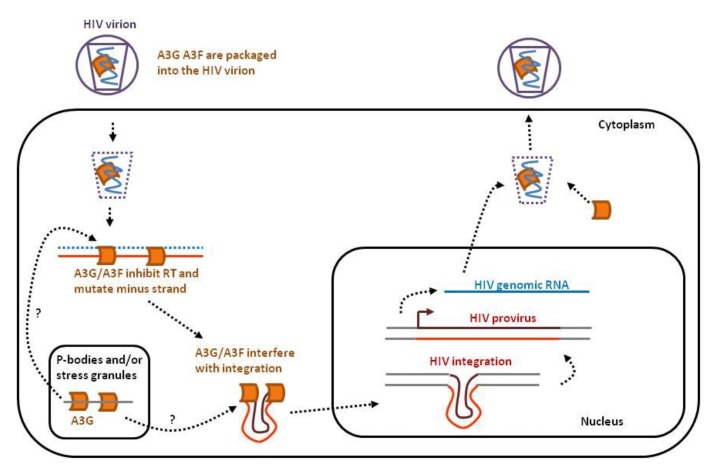
APOBEC3s interfere with several key steps of the HIV infectious cycle. After binding of the HIV virion to the cell membrane, the single-stranded RNA genome (**in blue**) is released into the cytoplasm together with APOBEC3G and 3F (**orange**). APOBEC3 proteins expressed by the host cell concentrate in P-bodies and stress granules. A3G and A3F inhibit reverse transcription, mutate viral DNA and perturb proviral integration into the host genome. In the absence of HIV Vif, A3G and A3F will be incorporated into the budding virions.

Historically, the first member of the APOBEC3 family was discovered in a groundbreaking study on HIV-1 [24]. A3G has indeed been shown to inhibit HIV infection and to be repressed by the viral Vif protein. Later on, a similar function was also attributed to other APOBEC3 proteins, namely A3DE, A3F and A3H [25,26,27]. Figure 1 illustrates the different mechanisms of HIV-1 inhibition by APOBEC3s. After binding of the HIV virion to the host cell membrane, the viral single-stranded RNA (ssRNA) genome is released into the cytoplasm and converted into double-stranded DNA (dsDNA) by reverse transcription. This dsDNA is then inserted into the host genome as an integrated provirus.

A3DE, A3F, A3G and A3H are expressed by CD4+ T cells upon HIV infection, are packaged into virions and lead to proviral DNA mutations [27]. A3G and A3F notably concentrate in cytoplasmic microdomains (non-membrane structures) called mRNA-processing bodies or P-bodies [28,29,30]. P-bodies are sites of RNA storage, translational repression and decay [31]. A3G exerts its anti-HIV effect mainly via its deaminase function inducing abundant and deleterious mutations within the HIV provirus, whereas A3F acts more preferentially through its deaminase-independent activity [32]. This deaminase-independent effect involves inhibition of reverse transcription priming and extension [14,15,16,17] and interference with proviral integration [21,22,23].

**Figure 2:**
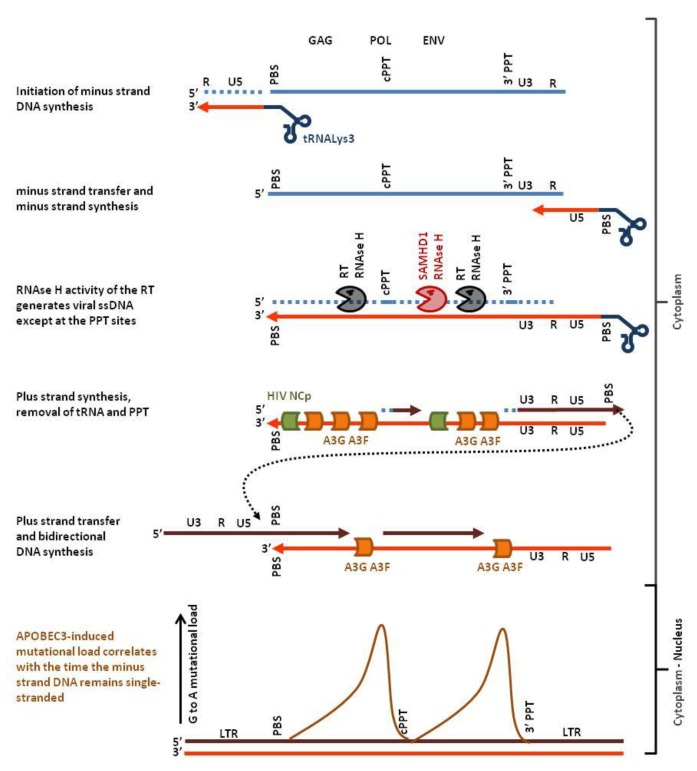
Hotspots of APOBEC3 editing in the HIV genome. Host cell tRNALys3 (**dark blue**) hybridizes to the primer binding sequence (PBS) of the single stranded plus-strand RNA genome (**light blue**) and initiates minus strand DNA synthesis (**light red**). After strand transfer, reverse transcription proceeds up to the PBS yielding minus-strand DNA. RNAse H then hydrolyses the RNA (**dotted light blue**) of the RNA:DNA hybrid leaving the minus-strand DNA single-stranded. APOBEC3 G and F (**orange**) have now access the ssDNA genome, deaminate deoxycytidine and inhibit plus strand DNA synthesis (**dark red**). RNAse H activity of SAMHD1 promotes exposure of the minus-strand DNA (red pacman) whereas HIV nucleocapsid (**green**) limits APOBEC3-edition. Deoxycytidine deamination of the minus strand generates G-to-A mutations on the plus strand. Since plus-strand DNA synthesis starts from the PolyPurine Tracts (cPPT, 3′PPT), ssDNA located distant to these sites will be accessible to APOBEC3 edition over a longer period of time. Therefore, the APOBEC3-related mutational load will also be higher (**brown curve**, schematic representation of the data from [33]).

APOBEC3-induced mutations are almost always G-to-A transitions of the plus-strand genetic code. Moreover, the mutation load is not homogeneous along the HIV provirus but presents two highly polarized gradients, each peaking just 5′ to the central polypurine tract (cPPT) and 5′ to the LTR (long terminal repeat) proximal polypurine tract (3′PPT) [33]. As illustrated in Figure 2, this mutational signature is due to the mechanism of HIV reverse transcription. Binding of the human tRNALys3 to the primer binding sequence (PBS) initiates the minus strand DNA synthesis by the virus-encoded reverse transcriptase protein (RT). The RT-associated ribonuclease H activity (RNAse H) selectively degrades the RNA strand of the RNA:DNA hybrid leaving the nascent minus-strand DNA free to hybridize with the complementary sequence at the 3′ end of the viral genomic ssRNA. After minus strand transfer, the viral RNA is reverse-transcribed into DNA. Whilst DNA synthesis proceeds, the RNAse H function cleaves the RNA strand of the RNA:DNA. Two specific purine-rich sequences (polypurine tracts cPPT and 3′PPT) that are resistant to RNAse H remain annealed with the nascent minus strand DNA. The reverse transcriptase uses the PPTs as primers to synthesize the plus-strand DNA. Finally, another strand transfer allows the production of the 5′ end of the plus-strand DNA (reviewed in [34]). From this complex multistep process, it appears that only the minus strand can be single-stranded (light red in Figure 2). Thus, G to A mutations observed on the plus strand (dark red in Figure 2) originate from C-to-T mutations on the minus strand. The gradient of mutational load actually correlates with the time that the minus strand remains single-chain [33].

To counteract inactivation, HIV-1 encodes the Vif protein that inhibits APOBEC3s. Vif prevents A3G, A3F and A3H from being packaged into the virion by recruitment to a cullin5-elonginB/C-Rbx2-CBFβ E3 ubiquitin ligase complex, resulting in their polyubiquitination and subsequent proteasomal degradation [35,36,37]. Other mechanisms can limit APOBEC3 access to the single-chain minus-strand DNA generated during reverse transcription. By stabilizing the viral core, the glycosylated Gag protein of the murine leukemia virus renders the reverse transcription complex resistant to APOBEC3 and to other cytosolic viral sensors [38]. The HIV nucleocapsid protein (NCp) is able to bind ssDNA in a sequence aspecific manner and prevents A3A from mutating genomic DNA during transient strand separation [39]. Degradation of the RNA strand from the RNA:DNA hybrid by the RNAse H activity of the reverse transcriptase contributes to expose the minus strand as a single-chain nucleic acid. Interestingly, the host factor SAMHD1 (sterile alpha motif and histidine-aspartic acid domain containing protein 1) restricts HIV via its RNAse H function, activity that may facilitate the access of the APOBEC3s to the transiently single-stranded minus strand [40,41].

#### 2.1.2. HTLV-1

Another human retrovirus, human T-lymphotropic virus 1 (HTLV-1), is also a target of A3G [42,43]. As in HIV-1 infection, A3G induces G-to-A transitions on the plus strand via deamination of deoxycytidines on the minus strand. HTLV-1 proviruses contain A3G-related base substitutions, including non-sense mutations [43]. Because HTLV-1 proviral loads mainly result from clonal expansion of infected cells, non-sense mutations are stabilized and amplified by mitosis, provided that viral factors stimulating proliferation are functional [44,45,46,47]. Although HTLV-1 does not seem to encode for a Vif-like protein, the frequencies of G-to-A changes in HTLV-1 proviruses are low, likely due to the mode of replication of HTLV-1 by clonal expansion [43,48]. This phenotype has also been associated with the ability of the viral nucleocapsid to limit A3G encapsidation [49].

#### 2.1.3. HERVs

Human endogenous retroviruses (HERV) are transposable elements which were evolutionary integrated into human lineage after infection of germline cells. HERVs are abundant in the human genome (about 8%) and exert important regulatory functions such as control of cellular gene transcription [50]. HERVs contain canonical retroviral *gag*, *pol* and *env* genes surrounded by two LTRs. Nevertheless, most HERVs are defective for replication because of inactivating mutations or deletions [51]. These mutations are likely associated with A3G activity because of a particular signature with a mutated C present in a 5′GC context instead of 5′TC for other APOBEC3s [52,53,54]. Interestingly, A3G is still able to inhibit a reconstituted functional form of HERV-K in cell culture [52].

#### 2.1.4. Simian Foamy Virus

SFV (simian foamy virus) is a retrovirus that is widespread among non-human primates and can be transmitted to humans [55]. A3F and A3G target SFV genome *in vitro*, leading to G-to-A transitions on the plus strand [56]. SFV genomes found in humans also display G-to-A mutations [57,58,59]. SFV codes for the accessory protein Bet, limiting APOBEC3 action [60,61,62,63].

### 2.2. Retroelements

**Figure 3:**
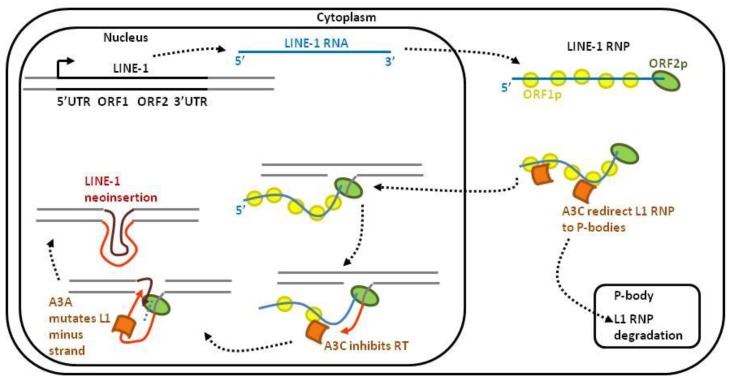
LINE-1 retrotransposons are targeted by APOBEC3s. After transcription, the LINE-1 mRNA is transported into the cytoplasm. After translation, the ORF1- and ORF2-encoded proteins associate with the LINE-1 RNA and form a ribonucleoprotein (RNP) complex. The LINE-1 RNP enters the nucleus, where the ORF2p endonuclease domain cleaves the chromosomal DNA. After cleavage, the 3′-hydroxyl is used by the LINE-1 reverse transcriptase to synthesize a cDNA of LINE-1. This target-site-primed reverse transcription typically results in the insertion of a 5′-truncated LINE-1 element into a new genomic location. Different APOBEC3s-dependent mechanisms control LINE-1 retrotransposition: (1) in the cytoplasm, A3C interacts with and redirects the L1-RNP into P-bodies for degradation; (2) in the nucleus, A3C inhibits reverse transcriptase processing while A3A mutates the minus strand LINE-1 DNA.

About half of the human genome is constituted by repetitive elements. Among them, non-LTR retroelements LINE-1 (long interspersed nuclear element-1), SINE (short interspersed nuclear elements) and Alu are capable of retrotransposition, *i.e.*, inserting a copy of themselves elsewhere in the genome. Since retrotranpositions can be harmful for genome integrity, these events are tightly controlled. In fact, only a small proportion of endogenous retroelements remains active in the germline cells because APOBEC3s protect the host genome from unscheduled retrotransposition (Figure 3). LINE-1 retrotransposition is initiated by transcription of a full-length LINE-1 RNA and translation of ORF1p and ORF2p. These two proteins associate with LINE-1 RNA to form the LINE-1 RiboNucleoProtein (L1 RNP) complex. Upon translocation of L1 RNP into the nucleus, LINE-1 is reverse transcribed and integrated into a new site of the host genome. A3C restricts LINE-1 retrotransposition in a deaminase-independent manner by redirecting and degrading the L1 RNP complex in P-bodies [20]. Within the nucleus, A3C also impairs LINE-1 minus strand DNA synthesis [20]. A3A prevents LINE-1 retrotransposition by deaminating the LINE-1 minus strand DNA [64]. Consistently, RNAse H treatment increases deamination of the LINE-1 minus strand [64].

### 2.3. Hepadnaviruses

**Figure 4:**
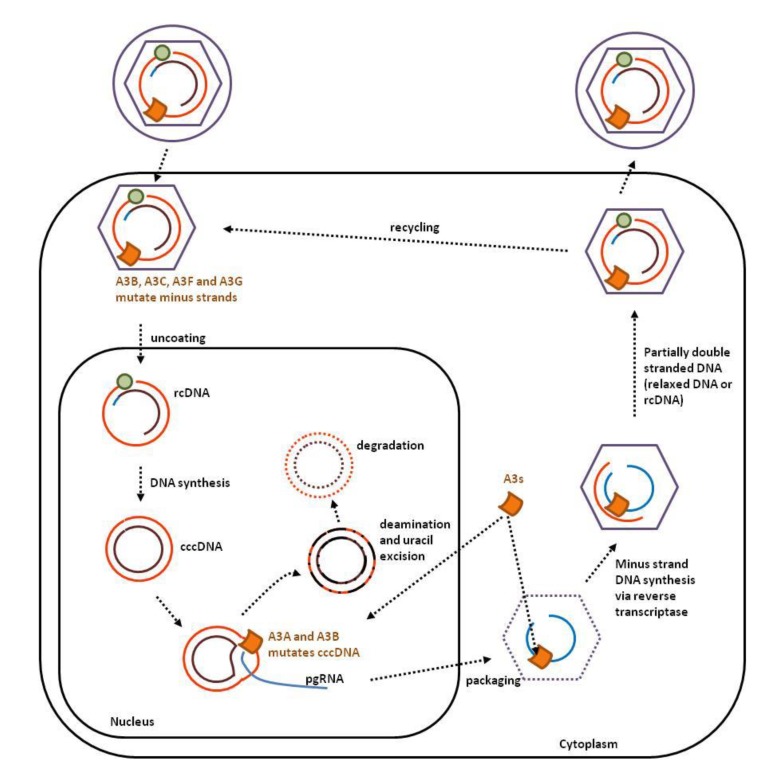
APOBEC3s interfere with several steps of the HBV replication cycle. The HBV viral particle contains a partially double-stranded DNA genome (relaxed circular DNA or rcDNA) that can be edited by A3G and A3F. Unlike HIV, HBV does not appear to encode Vif-like protein. Upon transfer into the nucleus, the plus strand of the rcDNA is replicated to form the covalently closed circular DNA genome (cccDNA). A3A and A3B deaminate the cccDNA genome leading to uracil excision and subsequent degradation.

Since their genome is partially single-strand, hepadnaviruses, such as human hepatitis B virus (HBV), are susceptible to APOBEC3 editing. Except A3DE, all APOBEC3s are able to edit the HBV genome *in vitro,* A3A being the most efficient [65,66]. APOBEC3 editing of HBV DNA has also been validated *in vivo* [67,68]. Since both minus and plus strands are susceptible to APOBEC3 editing, the mutational signature is more complex than in retroviruses [65,66,67]. HBV viral particles contain a partially double-stranded circular DNA genome (relaxed circular DNA or rcDNA; Figure 4). After uncoating of the viral particle, the rcDNA migrates into the nucleus, where minus-strand DNA synthesis is completed to generate the covalently closed circular double-stranded DNA genome (cccDNA).

In the nucleus, A3A and A3B deaminate HBV cccDNA (Figure 4). Since APOBEC3s require a ssDNA substrate, it is predicted that cccDNA melts during transcription. APOBEC3 deamination of deoxycytidine introduces deleterious mutations in the viral genome and initiates its catabolism via the uracil DNA glycosylase dependent pathway [12].

**Figure 5:**
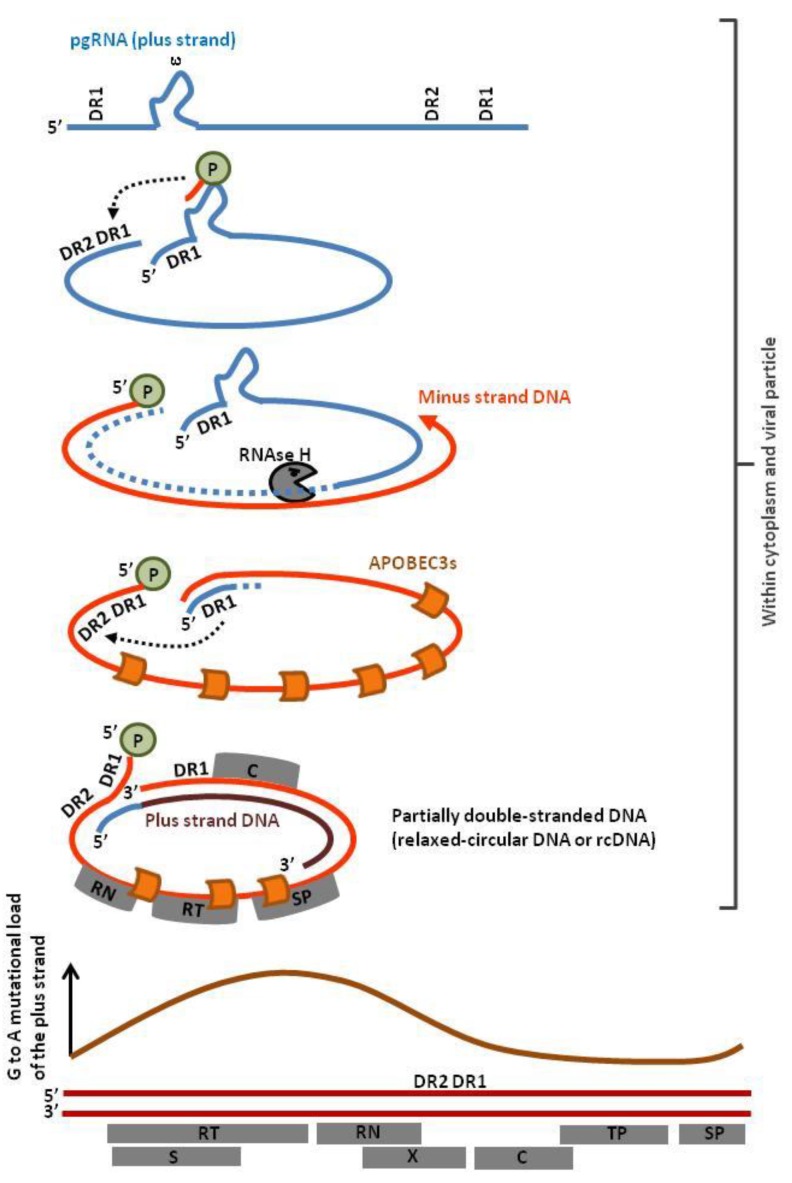
Profile of APOBEC3 editing of the HBV genome. The viral P protein initiates reverse transcription at the stem loop structure ε. The pregenomic RNA (pgRNA) contains two direct repeat sequences (DR1) at the 5′ and 3′ end of the viral genome, allowing strand transfer to the 5′ end of the viral genome. While synthesis of the minus-strand DNA proceeds, the RNAse H activity of the reverse transcriptase degrades the pgRNA except at the 5′ end. After a second strand transfer, the undigested pgRNA anneals with the direct repeat sequence DR2 and primes plus-strand gDNA synthesis, yielding relaxed circular DNA (rcDNA). Mutational load correlates with the time of exposure of ssDNA (**orange curve**, schematic representation of data extracted from reference [68]). Abbreviations within grey boxes read as follow: RT, Reverse Transcriptase; RN, RNAse; TP, Terminal Protein; SP, Spacer Domain; S, short surface gene; X, X gene, C, Core gene.

After transcription of the cccDNA, the pregenomic RNA (pgRNA) translocates into the cytoplasm and is reverse-transcribed into circular partially double-stranded DNA. This mechanism involves priming by the viral P protein, a strand transfer directed by DR1 annealing and degradation of the RNA template by the RNAse H activity of the reverse transcriptase (Figure 5, dotted light blue). The 5′ end of the pgRNA anneals with DR2, directs a second strand transfer and primes plus-strand DNA synthesis, yielding rcDNA. The minus strand DNA is deaminated proportionally to its exposure to APOBEC3s (Figure 5, orange curve) [68]. Since different subcellular compartments are involved (cytoplasm, nucleus, extracellular viral particles), multiple nuclear and cytoplasmic APOBEC3s (*i.e.*, A3A, A3B, A3C, A3F and A3G) edit the HBV genome [65].

Compared to HIV, additional APOBEC3 proteins (A3A, A3B and A3C) target the HBV genome in the nucleus. Incorporation of HIV into chromatin instead of an episome for HBV may protect the provirus from APOBEC3s editing by a mechanism involving Tribbles 3 proteins [69].

### 2.4. Herpesviruses

**Figure 6:**
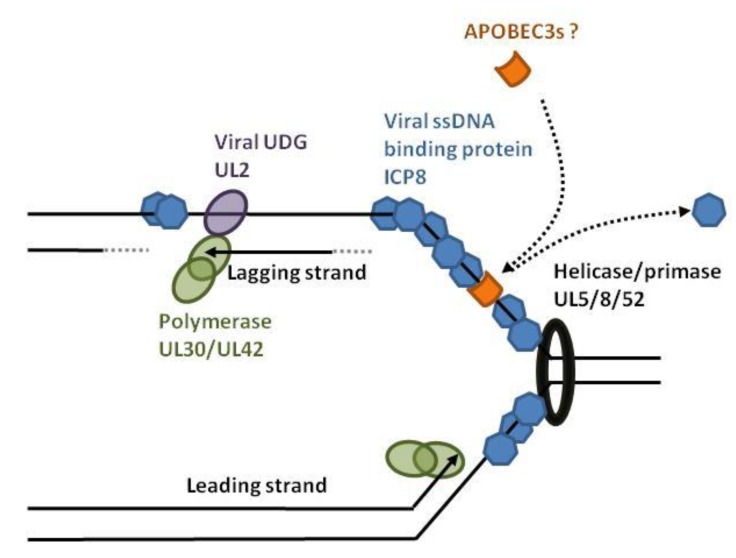
HSV-1 replication fork and hypothetical model of APOBEC3 editing. In the nucleus, replication of HSV-1 is initiated by the origin binding protein (UL9) that melts double-stranded DNA. The helicase/primase complex (UL5/UL8/UL52) unwinds and anneals RNA primers, allowing DNA replication by the UL30/UL42 complex. The viral protein ICP8 covers the transiently exposed single-stranded DNA and competes with the APOBEC3 binding. The viral UL2 is a uracil-DNA glycosylase (UDG) that favors replication-coupled base excision DNA repair.

Herpesviruses such as herpes simplex virus-1 (HSV-1) and Epstein-Barr virus (EBV) have a linear double-stranded DNA genome that is edited by APOBEC3 on both strands [70]. After infection, the HSV-1 capsid is transported to the nuclear pores and delivers the double-stranded linear DNA into the nucleus. After circularization of the viral genome, bidirectional DNA synthesis is initiated at the origins of replication [71,72]. This process requires DNA denaturation by the origin binding protein (UL9). The helicase/primase (UL5/UL8/UL52) and single-stranded DNA binding proteins (ICP8 coded by the UL29 gene) then associate with the origin of replication and recruit the DNA polymerase/UL42 complex (Figure 6). During DNA synthesis and transcription, nuclear APOBEC3s have access to single-stranded viral DNA. APOBEC3-edition of HSV-1 and EBV genomes is higher in the minus strand (G to A as opposed to C to T) [70]. It is hypothesized that, due to discontinued replication, the lagging strand exposes more viral ssDNA than the leading strand. HSV-1 and EBV encode orthologs of uracil-DNA glycosylases (UDG) that excise uridine at the replication fork. The HSV-1 UDG (UL2) binds to UL30, associates with the viral replisome and directs replication-coupled BER (base excision repair) to ensure genome integrity [73]. The viral UDG might therefore protect against APOBEC3 editing.

### 2.5. Papillomavirus

Human papillomaviruses (HPVs) are circular double-stranded DNA viruses. A3A, A3C and A3H are able to deaminate both strands of the 8Kb viral genome [74,75,76]. APOBEC3-edited HPV DNA is found in benign and precancerous cervical lesions [74]. Replication of the HPV genome occurs in the nucleus and is primarily based on the host replication machinery. The HPV protein E1 recruits ssDNA-binding protein RPA (replication protein A) during replication to cover the transiently exposed viral ssDNA [77].

### 2.6. TT Virus

Transfusion-transmitted virus (TTV) is a non-enveloped virus causing a persistent and asymptomatic infection. Having a circular single-stranded DNA genome, TTV is a prototypical substrate of APOBEC3s and shows APOBEC3-related mutations [78].

Together, these data show that viruses are targeted by particular isoforms of APOBEC3 depending on their modes of replication (Table 1) and have developed strategies to dampen ssDNA edition. A3G and A3F are restricted to the cytoplasm whereas A3A, A3B and A3C preferentially act in the nucleus. Importantly, the mutational load is proportional to the duration of single-stranded DNA exposure to APOBEC3s.

**Table 1:** Summary of the anti-viral activity of the different APOBEC3 isoforms. * It has been recently shown that A3A can also edit RNA transcripts [79].

	Sub-cellular localization	Substrate edited	Retro viruses				Retro elements	Hepadna viruses	Herpes viruses
			**HIV-1**	**HTLV-1**	**HERVs**	**SFV**			
**A3A**	cell wide	single stranded DNA, RNA *					+	+	+
**A3B**	nuclear	single stranded DNA						+	
**A3C**	cell wide	single stranded DNA					+	+	+
**A3DE**	cytoplasmic	single stranded DNA	+						
**A3F**	cytoplasmic	single stranded DNA	+			+		+	
**A3G**	cytoplasmic	single stranded DNA	+	+	+	+		+	
**A3H**	cell wide	single stranded DNA	+					+	+

## 3. Therapeutic Strategies by Perturbation of the Viral Mutation Rate

**Figure 7:**
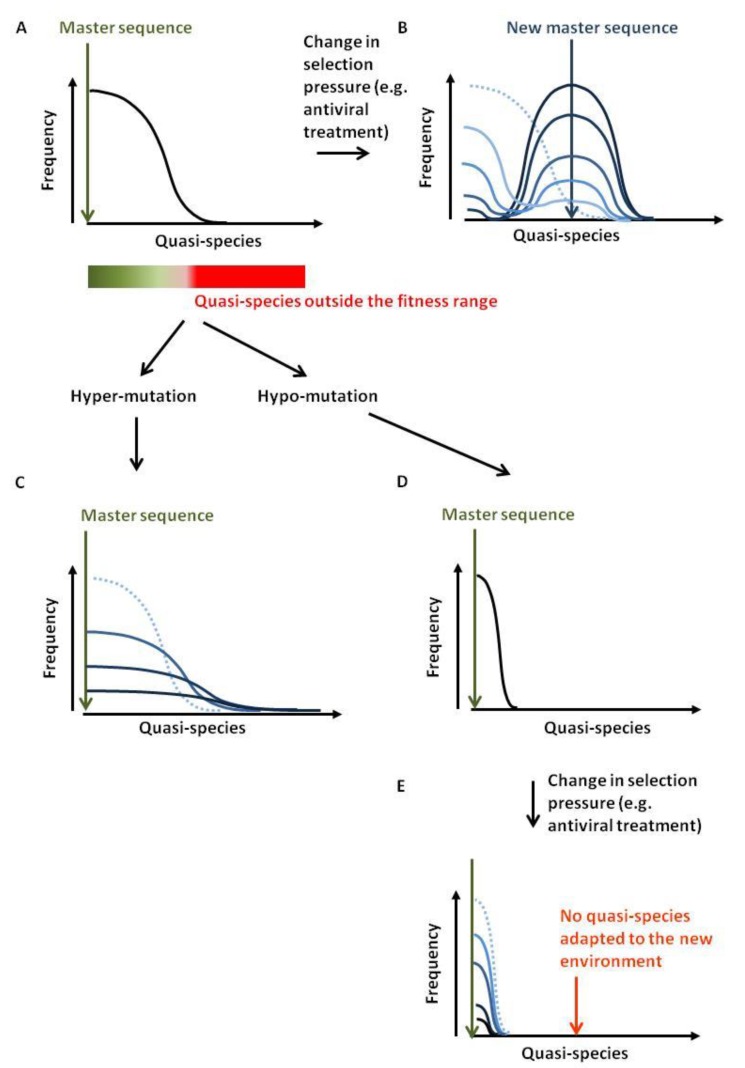
Antiviral strategy by hypo- or hyper-mutation. (**A**) Viral quasi-species refer to as a population of distinct but closely related viral genomes that only differ by a limited number of mutations. The frequency of these quasi-species spreads around a master sequence. The boundary of this population is dictated by the selection forces acting against the viral diversification. At equilibrium, quasi-species generated outside the fitness range will not persist. (**B**) If selection criteria are modified, the fittest sequence will change. If the original distribution contained this sequence, the population will first shrink and then re-grow around a new master sequence (from light to dark blue corresponding to the time evolution). (**C**) Excess of APOBEC3-directed mutations will affect fitness of the newly created quasi-species up to complete disappearance. (**D**,**E**) Hypo-mutation will restrict the range of quasi-species and limit adaptability to new environmental conditions.

Viral quasi-species refer to a population of distinct but closely related viral genomes that differ only by a limited number of mutations. The distribution of variants is dominated by a master sequence that displays the highest fitness within a given environment (Figure 7A). High mutation rates during viral replication are the driving force for quasi-species generation. Lethal mutations or inappropriate adaptation to the environmental conditions (e.g., anti-viral therapy, immune pressure) will clear unfit genomes. When conditions change, the fittest quasi-species may differ from the master sequence. Providing that the distribution contains an adequate variant, a new population will grow (Figure 7B). The rate of mutation and the selection pressure will dictate the wideness of the distribution. If the environmental changes are too drastic or the quasi-species distribution too narrow, the viral population will be unable to recover [80].

Emergence of quasi-species is thus a major issue that limits antiviral therapy. Viral populations can indeed accommodate environmental changes due to improved immunity (vaccination) or pharmacological inhibition. It is possible to affect quasi-species adaptability by modulating the frequencies of mutation [81,82].

The first approach, referred to as lethal mutagenesis or the hyper-mutation strategy, aims to introduce an excess of mutations in the viral genomes. If the mutational load per viral genome is too high, a substantial proportion of the new viruses will be defective or inadequately adapted to their environment. Introducing mutations in viruses would therefore decrease viral load (Figure 7C). In principle, exogenous induction of APOBEC3 expression could achieve this goal. This strategy has recently been exemplified for HBV, where forced expression of A3A and A3B induced HBV cccDNA hypermutation with no detectable effect on genomic DNA [12]. Nevertheless, this approach raises serious safety issues because APOBEC3 mutations could also drive cancer development [83,84,85]. Indeed, A3A and A3B over-expression in yeast creates mutational clusters and genomic rearrangements similar to those observed in human cancers, the mutational burden being magnified by DNA strand breaks [86,87,88,89]. Because the processing of double-strand break repair transiently exposes single-stranded nucleic acids, DNA repair could provide a substrate for nuclear deaminases. What would, for example, happen if an HBV-infected liver cell is being forced to express deaminases and at the same time has to repair DNA strand breaks generated by reactive oxygen species produced during alcohol catabolism [90]?

Therefore, it would be safer to promote hyper-mutation by targeting the viral factors that inhibit APOBEC3s. In that respect, Vif inhibitors are being developed [91,92]. Inhibition of viral ssDNA-binding proteins (like HSV-1 ICP8) might lead to increased access for endogenously expressed APOBEC3s to the viral ssDNA (Figure 6). Promotion of RNAse H activity during retrotranscription might facilitate the binding of the APOBEC3s to the viral ssDNA (Figure 2). Because reverse transcription is thought to start within the virion, promotion of APOBEC3s loading in to the viral particle will increase editing (MLV glyco-Gag shields the reverse transcription complex from APOBEC3 and cytosolic sensors [38]). Alternatively, it would be possible to target viral DNA repair mechanisms (e.g., via inhibitors against the viral UDG UL2 of HSV-1, Figure 6). In these cases, safety issues are related to the emergence of sub-lethal APOBEC3-mutations and promotion of drug resistant quasi-species.

The reverse strategy would be to reduce mutation rate by inhibiting APOBEC3, thereby narrowing the quasi-species spectrum and limiting viral adaptability to new environmental conditions (Figure 7D,E). APOBEC3s inhibitors are currently being developed and evaluated [93,94]. This approach, which paradoxically targets a well-conserved mechanism of mammalian innate immunity, would preserve host genome integrity. Potential risks of this therapy pertain to adequate control of endogenous retroelements and opportunistic infections.

## 4. Conclusions

Single-strand DNA editing by APOBEC3 proteins is a very powerful mechanism of mammalian innate immunity that mutates and inactivates viral genomes. The outcome of infection is the result of a finely tuned balance between onset of mutations, generation of quasi-species and APOBEC inhibition by viral factors. Understanding the mechanisms involved reveals new prospects for therapeutic strategies that interfere with APOBEC3 deamination of cytosine residues in nascent viral DNA.
